# Taxonomic review of the genus *Dirrhagofarsus* in Korea (Coleoptera, Eucnemidae)

**DOI:** 10.3897/zookeys.781.22335

**Published:** 2018-08-13

**Authors:** Jinbae Seung, Jyrki Muona, Seunghwan Lee

**Affiliations:** 1 Insect Biosystematics Laboratory, Department of Agricultural Biotechnology, Seoul National University, Seoul 151-921, Republic of Korea; 2 Research Institute for Agricultural and Life Sciences, Seoul National University, Seoul 151-921, Republic of Korea; 3 Finnish Museum of Natural History, Zoological Museum, FIN-00014 University of Helsinki, Finland

**Keywords:** *
Dirrhagofarsus
*, Eucnemidae, Korea, new combination, taxonomy

## Abstract

The genus *Dirrhagofarsus* is firstly recorded from Korea with three species: *Dirrhagofarsuslewisi* (Fleutiaux, 1900), *Dirrhagofarsusmodestus* (Fleutiaux, 1923), and *Dirrhagofarsusunicolor* (Hisamatsu, 1960). A key to Korean species of *Dirrhagofarsus*, with diagnoses, redescriptions, and photographs of important structures is provided. In this work, Dirrhagusmodestusf.unicolor Hisamatsu, 1960 is regarded as a valid species, *Dirrhagofarsusunicolor* (Hisamatsu, 1960), **comb. n.**

## Introduction

The genus *Dirrhagofarsus* was originally characterized by strongly convex elytral apices, dilate tarsomere IV and shiny notosternal antennal grooves (Fleutiaux 1935). Ford and Spilman (1979) described the biology and larval features of the type species, *Dirrhagofarsuslewisi* (Fleutiaux) adding a new diagnostic feature, the lateral frontal carinae. Muona (1993) transferred two species to *Dirrhagofarsus*: *Hypocaelusattenuatus* Mäklin, 1845 and *Dirrhagusmodestus* Fleutiaux, 1923. Subsequently, Otto et al. (2014) described *Dirrhagofarsusernae* from North America. Also, Otto (2016) described *Dirrhagofarsusfoveicollis* from Laos. Finally, Kovalev (2016) transferred *Dirrhagusferrugineus* Reitter, 1889 to *Dirrhagofarsus*. Thus, genus *Dirrhagofarsus* included six species worldwide (Muona 2007; Otto et al. 2014; Otto 2016; Kovalev 2016).

Hisamatsu (1960) described what he considered a light-coloured form of *Dirrhagusmodestus* as Dirrhagusmodestusf.unicolor. After that, Muona and Alaruikka (2007) commented that f.unicolor was proposed as infrasubspecific name and omitted it from their catalogue. However, JM studied four such specimens collected in Japan (Fukushima Pref., Fukushima City, Moniwa, 1976-06-19, male and two females; Fukushima Pref., Mt. Asahi, 1974-07-29; S. Ohmomo leg.) and observed that they were a distinct species. Although the holotype has not been studied, Hisamatsu (1960) provided excellent images of the characteristic aedeagus, and illustrations of all other features are also as he described. Close to that time, JS and SL discovered an apparently new *Dirrhagofarsus* species from Korea. After discussion, the authors concluded that the Korean and Japanese forms were identical. Species names given to infrasubspecific forms are usually unavailable; however, there are exceptions to this. If such names are proposed before 1 January 1961 (ICZN, article 10.2), they are available with the original authority unless the description includes information showing that the author intended it an infrasubspecific grouping. This is not the case with Hisamatsu (1960) and thus the name of this previously ignored species becomes *Dirrhagofarsusunicolor* (Hisamatsu, 1960), comb. n., stat. n.

Herein, we firstly report and review Korean species of genus *Dirrhagofarsus*, including three species: *Dirrhagofarsuslewisi* (Fleutiaux, 1900), *D.modestus* (Fleutiaux, 1923), *D.unicolor* (Hisamatsu, 1960). A key to Korean species of *Dirrhagofarsus*, with diagnosis, redescriptions, and photographs is provided.

## Materials and methods

Most samples were collected using flight intercept traps, light trapping, or by hand during 2015 and 2016. Samples were preserved in 95% ethanol and made into dried specimens by the double mounted method (pinned with a micropin to a block of cork, which is mounted on a standard insect pin). In order to examine detailed structures, some specimens were softened in distilled water for an hour and dissected using a micro-pin and forceps. Photographs for each species were taken using a digital camera (Canon EOS-600D) through MP-E 65mm lens. Samples for this study are deposited in the insect collection of the College for Agriculture and Life Sciences, Seoul National University (**CALS**, SNU, Seoul, Korea).

Morphological terminology follows Muona (1993) and Otto (2016). We measured the length of the pronotum, from the anterior edge of the pronotum to the apex of pronotal posterior angle.

We identified the species by literature comparison (Fleutiaux 1900, 1923, 1935; Hisamatsu 1960, 1985; Otto et al. 2014).

## Results

### Family Eucnemidae Eschscholtz, 1829

#### Subfamily Melasinae Fleming, 1821

##### Tribe Dirhagini Reitter, 1911

###### Genus Dirrhagofarsus

Taxon classificationAnimaliaColeopteraEucnemidae

Fleutiaux, 1935

Dirrhagofarsus Fleutiaux, 1935: 15. Type species: Microrhaguslewisi Fleutiaux, 1900.

####### Diagnosis.

Head: vertex with transverse row of dense vestiture; frons with a pair of longitudinal carinae near compound eyes; antennae subfiliform to serrate; antennomere II shorter than IV. Prothorax: pronotum parallel-sided, about as long as wide; lateral carina divided into anterior and posterior parts; antennal grooves notosternal, parallel-sided, with lateral marginal carina. Pterothorax: elytra with strongly convex apices in lateral view; mesepimeron fused with mesepisternum; metepisternum narrow, subparallel-sided, 7–9 × longer than wide; metacoxal plate strongly expanded medially. Leg: tibiae and tarsi slender; metatarsomere I 1.5 × longer than II–IV combined; tarsal claws simple. Abdomen: ventrites connate, ventrite V sinuate and acute in ventral profile. Aedeagus: dorsoventrally compressed; median lobe bifurcate at apex; lateral lobes not fused with ventral plate, slender, narrowing apically (Fleutiaux 1935; Muona 2000, 2011; Otto et al. 2014).

###### Key to species of Korean *Dirrhagofarsus*

**Table:** 

1	Antennomere III of male less than 1.5 × longer than IV; elytra 2.65–2.70 × longer than combined width	**2**
–	Antennomere III of male more than 1.5 × longer than IV (Fig. 3E); elytra 2.5 × longer than combined width (Fig. 3A)	***D.unicolor* (Hisamatsu), comb. n.**
2	Frons without medio-longitudinal carina (Fig. 1E); elytra with strongly convex apices in lateral view, apices pointed and raised above ventrite V (Fig. 1C)	***D.lewisi* (Fleutiaux)**
–	Frons with weak medio-longitudinal carina (Fig. 2G); elytra with simply convex apices in lateral view, apices blunt and contact with ventrite V (Fig. 2D)	***D.modestus* (Fleutiaux)**

####### Dirrhagofarsus lewisi

Taxon classificationAnimaliaColeopteraEucnemidae

(Fleutiaux, 1900)

Figure 1

Microrhagus lewisi Fleutiaux, 1900: 358.
Dirrhagus lewisi Fleutiaux, 1923: 308.
Dirrhagofarsus lewisi Fleutiaux, 1935: 16.

######## Diagnosis.

Body: mostly coloured dark brown. Head: frons simple, without medio-longitudinal carina; anterior edge of frontoclypeal region 2.7 × wider than distance between antennal sockets in female; antennomere III 1.3 × longer than IV in female. Pronotum: anterolateral carina one-fifth as long as pronotum; posterolateral carina four-fifths as long as pronotum. Pterothorax: elytra 2.65 × longer than combined width, apices with strongly convex apices in lateral view. Leg: metatarsomere II 1.6 × longer than III, as long as V.

######## Redescription.

**Figure 1.**
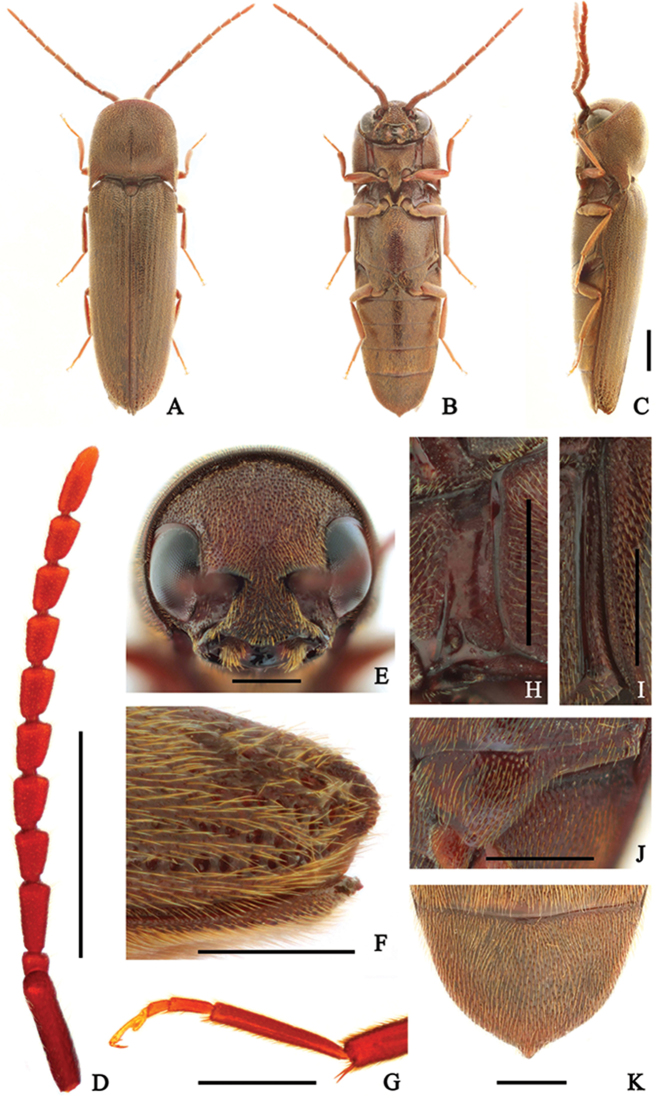
*Dirrhagofarsuslewisi* (Fleutiaux, 1900). female. **A** dorsal habitus; **B** ventral habitus **C** lateral habitus **D** antenna **E** frons **F** elytral apex in lateral view **G** metatarsus **H** hypomeron **I** metepisternum **J** metacoxal plate **K** abdominal ventrite V. Scale bar: 1 mm (**A–D**); 0.5 mm (**E–K**).

**Female** (Fig. 1A–C) 6.1–7.7 mm long and 1.7–2.2 mm wide. **Body** brown to dark brown; antennae and legs red-brown; surface weakly glossy, covered with yellow-brown pubescence. **Head** deeply inserted into prothorax, barely visible in dorsal view; surface coarse, with circular, irregularly sized and spaced punctures, more rugose near occiput and frontoclypeal region; frons simple, without medio-longitudinal carina; frontoclypeal region (Fig. 1E) slightly depressed at base, obtusely trilobate at anterior edge, anterior edge 2.7 × wider than distance between antennal sockets. **Antennae** (Fig. 1D) weakly serrate, almost reaching abdominal ventrite II, with yellow-grey pubescence; antennomere II conical and shortest; antennomere III 2.5 × longer than II, and 1.3 × longer than IV; antennomeres IV–X subequal, slightly shortened apically; antennomere XI 2.9 × longer than wide, and 1.5 × longer than X. **Pronotum** as long as wide and obtusely arcuate anteriorly; surface with finer, more regularly sized and regularly spaced punctures than on head, especially anteriorly; disc with a medio-longitudinal carina at basal half; anterolateral carina one-fifth as long as pronotum; posterolateral carina four-fifths as long as pronotum; antescutellar lobe obtusely notched; pronotal posterior angles acute, exceeding posterior edge of antescutellar lobe. **Scutellum** straight anteriorly and evenly arcuate behind anterolateral angles, 1.1 × wider than long; surface rough, sparsely pubescent. **Elytra** 2.65 × longer than combined width, parallel-sided in dorsal view, and attenuate near apices; disc weakly striate, with shallow, irregularly sized and spaced punctures; several large, deep punctures present near apices; apices strongly convex in lateral view, apices pointed and raised above ventrite V (Fig. 1F). **Prosternum** wider than long, slightly widened anteriorly; punctures finer and more regularly spaced than on head; prosternal process gradually tapered, and curved dorsally posteriorly; hypomeron with coarse surface, with larger punctures than on prosternum; antennal grooves (Fig. 1H) well-developed, notosternal, parallel-sided, with lateral marginal carina, non-punctate. **Mesoventrite** with coarse surface. **Metaventrite** with punctures denser than on prosternum; with a weak median groove along length of metaventrite; metepisternum (Fig. 1I) slightly widened posteriorly, widest part 1.7 × wider than outer edge of metacoxal plate; metacoxal plate (Fig. 1J) medially four × longer than laterally. **Legs** (Fig. 1G) with metatarsomere II 1.6 × longer than III, as long as V. **Abdomen** with denser punctures than on metaventrite (Fig. 1K). **Male**. Not examined.

######## Specimens examined.

**Gyeonggi-Do** 1♀, Mt. Bara, Hagui-dong, Uiwang-si, N37°22.34', E127°1.37', 189m alt., light trap, 22 June 2015, J. B. Seung leg. (SNU); **Gangwon-Do** 1♀, Beopheung-ri, Suju-myeon, Yeongwol-gun, N37°22.69', E128°15.26', 550m alt., flight intercept trap, 03–16 July 2015, leg. Seung and Lee leg. (SNU); 1♀, Seorim-ri, Seo-myeon, Yangyang-gun, N37°56.66', E128°31.17', 292m alt., 09 July 2016, S. H. Lee leg. (SNU); **Jeollanam-Do** 1♀, Jungdae-ri, Ganjeon-myeon, Gurye-gun, N35°6.44', E127°35.90', 668m alt., flight intercept trap, 04–15 July 2016, Seung and Lee leg. (SNU).

######## Distribution.

Korea (New record), Japan, Nearctic Region (USA).

######## Remarks.

A female individual of *Dirrhagofarsuslewisi* is observed under bark of rotten fallen tree. Additionally, they were rarely collected at light traps. They were observed clicking as well as flying and running.

####### Dirrhagofarsus modestus

Taxon classificationAnimaliaColeopteraEucnemidae

(Fleutiaux, 1923)

Figure 2

Dirrhagus modestus Fleutiaux, 1923: 308.
Rhacopus modestus Hisamatsu, 1985: 50.
Dirrhagofarsus modestus Muona, 1993: 46.

######## Diagnosis.

Body: mostly coloured black. Head: frons with a weak medio-longitudinal carina; anterior edge of frontoclypeal region 2.9 × wider than distance between antennal sockets in male, 2.7 × wider in female; antennomere III 1.35 × longer than IV in male, 1.7 × longer in female. Pronotum: anterolateral carina one-sixth as long as pronotum; posterolateral carina four-fifths as long as pronotum. Pterothorax: elytra 2.7 × longer than combined width, apices with fairly convex apices in lateral view. Leg: metatarsomere II 1.6 × longer than III, as long as V. Aedeagus: 5.3 × longer than wide; lateral lobes as long as median lobe, phallobase trapezoidal, one-sixth as long as aedeagus.

######## Redescription.

**Figure 2.**
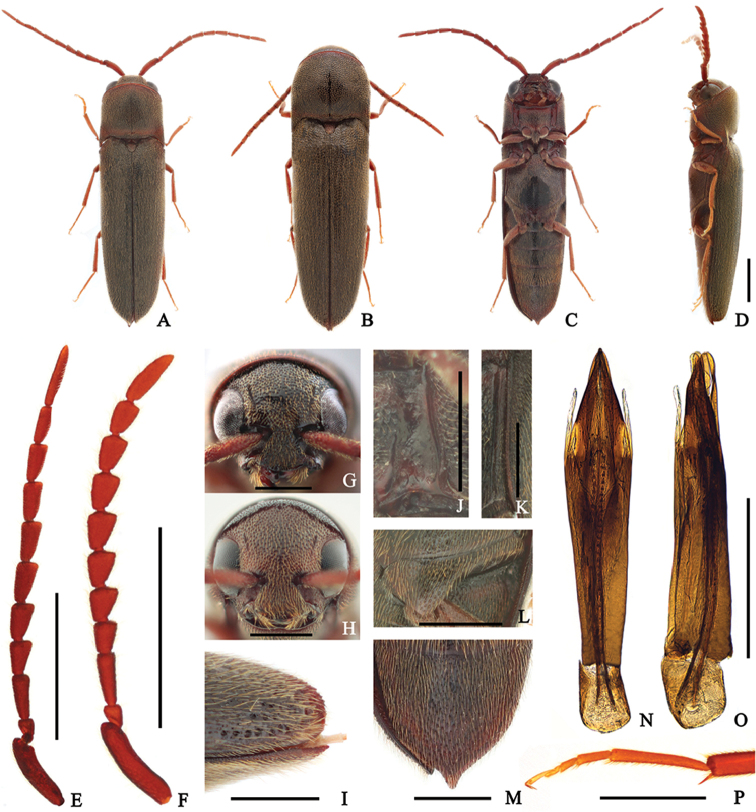
*Dirrhagofarsusmodestus* (Fleutiaux, 1923). **A, C–E, G, J–P** male **B, F, H, I** female **A–B** dorsal habitus **C** ventral habitus **D** lateral habitus **E–F** antenna **G–H** frons **I** elytral apex in lateral view **J** hypomeron **K** metepisternum **L** metacoxal plate **M** abdominal ventrite V **N–O** aedeagus **P** metatarsus. Scale bar: 1 mm (**A–F**); 0.5 mm (**G–P**).

**Male** (Fig. 2A, C–D) 4.5–5.9 mm long and 1.2–1.5 mm wide. **Body** black; antennae, mouthparts, anterior and posterior edge of pronotum red-brown; tibiae and tarsi brown to red-brown; surface shiny, covered with yellow-brown pubescence. **Head** deeply inserted into prothorax; surface coarse, with circular, irregularly sized and spaced punctures, rugose and more irregular near frontoclypeal region; frons with a weak medio-longitudinal carina; frontoclypeal region (Fig. 2G) weakly depressed at base, obtusely rounded at anterior edge, anterior edge 2.9 × wider than distance between antennal sockets. **Antennae** (Fig. 2E) weakly serrate, almost reaching abdominal ventrite II, with yellow-brown pubescence; antennomere II conical and shortest; antennomere III rectangular, 2.5 × longer than wide, two × wider than II, and 1.35 × longer than IV; antennomeres IV–X gradually lengthened and narrowed apically; antennomere XI 5.5 × longer than wide, and 1.7 × longer than X. **Pronotum** as long as wide and rounded anteriorly; surface with finer and denser punctures than on head, gradually more rugose laterally; disc with a medio-longitudinal carina at basal half; anterolateral carina one-sixth as long as pronotum; posterolateral carina approximately four-fifths as long as pronotum. **Scutellum** with straight anterior edge, gradually narrowed posteriorly with rounded apex; surface rough, sparsely pubescent. **Elytra** 2.7 × longer than combined width; disc weakly striate, with shallow, scattered punctures on intervals; several large, deep punctures present near apices; apices simply convex in lateral view in both sexes, apices blunt and contact with ventrite V (Fig. 2I). **Prosternum** slightly wider than long, parallel-sided; punctures more regularly spaced than on head, finer and denser at anterior and posterior regions; prosternal process gradually tapered and curved dorsally posteriorly; hypomeron with coarse surface, more irregularly sized than on prosternum; antennal grooves well-developed, notosternal, parallel-sided, with lateral marginal carina, barely punctate, and glabrous (Fig. 2J). **Mesoventrite** with rough surface. **Metaventrite** with finer, sparser, punctures than on prosternum, especially at middle; metepisternum (Fig. 2K) parallel-sided, width of posterior edge as wide as outer edge of metacoxal plate; metacoxal plate (Fig. 2L) medially four × longer than laterally. **Legs** (Fig. 2P) with metatarsomere II 1.6 × longer than III, as long as V. **Abdomen** with finer punctures than on metaventrite (Fig. 2M). **Aedeagus** (Fig. 2N–O) 5.3 × longer than wide; median lobe almost straight, gradually narrowed distally, deeply bifurcate at apex; endophallus reaching basal piece; lateral lobes as long as median lobe, with basally attached secondary lateral lobes; secondary lateral lobes slender, subparallel-sided, weakly pointed apically; phallobase trapezoidal, 1.25 × longer than wide and one-sixth as long as aedeagus.

######## Sexual dimorphism.

**Female** (Fig. 2B) can be distinguished from male by following characters: body larger and stouter, 5.2–6.8 mm long, 1.4–1.9 mm wide; base of frontoclypeal region wider, anterior edge 2.7 × wider than distance between antennal sockets (Fig. 2H); antennae (Fig. 2F) relatively shorter, almost reaching metacoxal plate; antennomere III 1.7 × longer than IV; antennomeres IV–X stouter; antennomere XI 3.3 × longer than wide.

######## Specimens examined.

**Seoul-Si** 7♂9♀, Mt. Gwanak, Daehak-dong, Gwanak-gu, Seoul-si, N37°27.06', E126°56.82', 184m alt., 18 January 2016, J. B. Seung leg. (collected in overwintering larval stage, 03. iv. 2016, adult emergence) (SNU); **Gyeonggi-Do** 1♀, Mt. Bara, Hagui-dong, Uiwang-si, N37°22.34', E127°1.37', 189m alt., light trap, 22 June 2015, J. B. Seung leg. (SNU); 2♂1♀, Mt. Bara, Hagui-dong, Uiwang-si, N37°22.38', E127°1.34', 174m alt., light trap, 01 June 2016, J. B. Seung leg. (SNU); **Gangwond-Do** 1♂, Beopheung-ri, Suju-myeon, Yeongwol-gun, N37°22.69', E128°15.26', 550m alt., flight intercept trap, 19 June–02 July 2015, Seung and Lee leg. (SNU); 1♀, Deokgu-ri, Sangdong-eup, Yeongwol-gun, N37°5.57', E128°48.99', 648m alt., flight intercept trap, 19 June–02 July 2015, Seung and Lee leg. (SNU); 1♀, Beopheung-ri, Suju-myeon, Yeongwol-gun, N37°22.69', E128°15.26', 550m alt., flight intercept trap, 03–16 July 2015, leg. Seung and Lee leg. (SNU); 1♀, Hoenggye-ri, Daegwanryeong-myeon, Pyeongchang-gun, N37°40.84', E128°45.78', 902m alt., flight intercept trap, 05–29 June 2016, Seung and Jung leg. (SNU); 3♀, Suha-ri, Daegwanryeong-myeon, Pyeongchang-gun, N37°36.60', E128°43.19', 803m alt., flight intercept trap, 05–29 June, 2016, Seung and Jung leg. (SNU); **Jeollanam-Do** 2♀, Jungdae-ri, Ganjeon-myeon, Gurye-gun, N35°6.44', E127°35.90', 668m alt., flight intercept trap, 04–15 July 2016, Seung and Lee leg. (SNU); **Jeju-Do (Is.)** 2♂, Gyorae gotjawal, Gyorae-ri, Jocheon-eup, Jeju-si, N33°26.35', E126°40.21', 428m alt., flight intercept trap, 10 June–21 July 2016, Seung and Jung leg. (SNU).

######## Distribution.

Korea (New record), Japan, Russia (Far East).

######## Remarks.

Mature larvae of *Dirrhagofarsusmodestus* were observed in U-form in oval larval cells in standing dead *Alnusjaponica* (Thunb.) Steudel (Fagales, Betulaceae) in January. Adults emerged at the same time as eucnemid species, *Dirrhagofarsusunicolor* and *Hylis* sp. 70 days later following rearing at room temperature.They were commonly collected at light traps. They were observed clicking as well as flying and running.

####### Dirrhagofarsus unicolor

Taxon classificationAnimaliaColeopteraEucnemidae

(Hisamatsu, 1960), comb. n. stat. n.

Figure 3

Dirrhagus modestus f. unicolor Hisamatsu, 1960: 102.

######## Diagnosis.

Body: mostly coloured brown. Head: frons simple, without medio-longitudinal carina; anterior edge of frontoclypeal region 3 × wider than distance between antennal sockets in male, 2.8 × wider in female; antennomere III 1.5 × longer than IV in male, 1.75 × longer in female. Pronotum: anterolateral carina one-sixth as long as pronotum; posterolateral carina four-fifths as long as pronotum. Pterothorax: elytra 2.5 × longer than combined width, apices with weakly convex apices in lateral view. Leg: metatarsomere II 1.3 × longer than III, metatarsomere V 1.2 × longer than II. Aedeagus: 4.5 × longer than wide; lateral lobes slightly longer than median lobe; phallobase rectangular, almost one-fifth as long as aedeagus.

######## Redescription.

**Figure 3.**
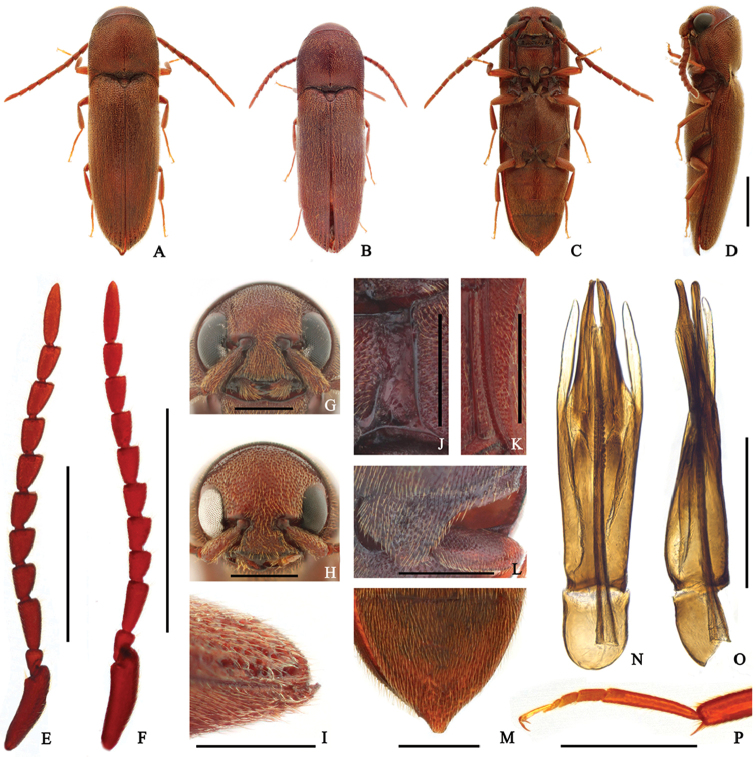
*Dirrhagofarsusunicolor* (Hisamatsu, 1960). **A, C–E, G, I–P** male **B, F, H** female. **A–B** dorsal habitus **C** ventral habitus **D** lateral habitus **E–F** antenna **G–H** frons **I** elytral apex in lateral view **J** hypomeron **K** metepisternum **L** metacoxal plate **M** abdominal ventrite V **N–O** aedeagus **P** metatarsus. Scale bar: 1 mm (**A–F**); 0.5 mm (**G–P**).

**Male** (Fig. 3A, C–D) 4.3–5.3 mm long and 1.2–1.5 mm wide. **Body** brown with yellow-brown tarsi; surface moderately glossy, covered with golden pubescence. **Head** moderately inserted into prothorax; surface with circular and regularly sized punctures, denser near frontoclypeal region; frons simple, without medio-longitudinal carina; frontoclypeal region (Fig. 3G) weakly depressed at base, feebly trilobate at anterior edge, anterior edge 3 × wider than distance between antennal sockets. **Antennae** (Fig. 3E) serrate, almost reaching metacoxal plate, with yellow-brown pubescence; antennomere II conical and shortest; antennomere III rectangular, 2.3 × longer than wide, 2 × longer than II, and 1.5 × longer than IV; antennomeres IV–X subequal, gradually narrowed apically; antennomere XI 3.8 × longer than wide, and 1.7 × longer than X. **Pronotum** as long as wide and arcuate anteriorly; surface rougher than head; disc weakly depressed at middle; with a short median carina at base; anterolateral carina one-sixth as long as pronotum; posterolateral carina four-fifths as long as pronotum. **Scutellum** triangular, 1.3 × wider than long, gradually narrowed posteriorly to slightly rounded posterior edge; surface coarse, densely pubescent. **Elytra** 2.5 × longer than combined width; disc barely striate, with irregularly sized and spaced punctures; several large, deep punctures present near apices; apices weakly compressed and simply rounded near sutural region in lateral view (Fig. 3I). **Prosternum** wider than long, parallel-sided; surface with punctures like as on head, slightly larger laterally; prosternal process gradually tapered and curved dorsally posteriorly; hypomeron with coarse surface, with punctures more irregularly sized than on prosternum; with deep pore at posterior fossae; antennal grooves (Fig. 3J) well-developed, notosternal, parallel-sided, with lateral marginal carina, barely punctate, and glabrous. **Mesoventrite** with coarse surface, with irregularly sized and spaced punctures. **Metasventrite** with punctures like as on prosternum, slightly larger and denser laterally; median groove present, not reaching anterior edge; metepisternum (Fig. 3K) slightly widened posteriorly, and widest part 1.5 × wider than outer edge of metacoxal plate; metacoxal plate (Fig. 3L) medially four × longer than laterally. **Legs** (Fig. 3P) with metatarsomere II 1.3 × longer than III; metatarsomere V 1.2 × longer than II; claws simple. **Abdomen** with denser punctures than on metaventrite (Fig. 3M). **Aedeagus** (Fig. 3N–O) 4.5 × longer than wide; median lobe almost straight, gradually narrowed distally, deeply and narrowly bifurcate apically; endophallus reaching basal piece; lateral lobes slightly longer than median lobe, slightly curved ventrally, with basally attached secondary lateral lobes; secondary lateral lobes subparallel-sided, weakly pointed apically; phallobase rectangular, almost one-fifth as long as aedeagus.

######## Sexual dimorphism.

**Female** (Fig. 3B) can be distinguished from male by following characters: body slightly stouter, 4.5–5.7 mm long and 1.3–1.7 mm wide; base of frontoclypeal region slightly wider, anterior edge 2.8 × wider than distance between antennal sockets (Fig. 3H); antennomere III 1.75 × longer than IV; antennomeres IV–X stouter (Fig. 3F).

######## Specimens examined.

**Seoul-Si** 4♂, Mt. Gwanak, Daehak-dong, Gwanak-gu, Seoul-si, N37°27.06', E126°56.82', 184m alt., 18 January 2016, J. B. Seung leg. (collected in overwintering larval stage, 03. iv. 2016, adult emergence) (SNU); **Gyeonggi-Do** 2♂, Mt. Bara, Hagui-dong, Uiwang-si, N37°22.34', E127°1.37', 189m alt., light trap, 22 June 2015, J. B. Seung leg. (SNU); 1♂, Mt. Bara, Hagui-dong, Uiwang-si, N37°22.38', E127°1.34', 174m alt., light trap, 01 June 2016, J. B. Seung leg. (SNU); 1♂, Mt. Bara, Hagui-dong, Uiwang-si, N37°22.38', E127°1.34', 174m alt., light trap, 04 June 2016, M. S. Oh leg. (SNU); 2♀, Baekgok-ri, Mado-myeon, Hwaseong-si, N37°10.65', E126°43.64', 115m alt., flight intercept trap, 06–28 June 2016, Seung and Yeom leg. (SNU).

######## Distribution.

Korea (New record), Japan.

######## Remarks.

Last instar larvae of *Dirrhagofarsusunicolor* were collected in standing dead *A.japonica* trees in January. They remained in U-form in oval larval cells. Adults emerged together with other eucnemid species, *Dirrhagofarsusmodestus* and *Hylis* sp., 70 days later following rearing at room temperature. Most of specimens were collected by light trap, occasionally by flight intercept traps. They were observed clicking as well as flying and running.

## Supplementary Material

XML Treatment for Dirrhagofarsus

XML Treatment for Dirrhagofarsus lewisi

XML Treatment for Dirrhagofarsus modestus

XML Treatment for Dirrhagofarsus unicolor
